# Effectiveness of a Breast Cancer Educational Conference Targeting Healthcare Workers in Honduras

**DOI:** 10.1155/ijbc/1855494

**Published:** 2024-12-21

**Authors:** Saranya Prathibha, Mario Zuniga, Suyapa Bejarano, Flora Duarte, Merlin Antunez, Alejandra Z. Molina, Noelle Hoven, Schelomo Marmor, Jennifer Witt, Jane Hui, Todd M. Tuttle

**Affiliations:** ^1^Department of Surgery, University of Minnesota, Minneapolis, Minnesota, USA; ^2^Department of Surgery, One World Surgery, Honduras; ^3^Department of Medical Oncology, La Liga Cancer Center, San Pedro Sula, Honduras; ^4^Department of Medical Oncology, Emma Romero Cancer Center, Tegucigalpa, Honduras; ^5^Department of Medical Oncology, Hospital San Felipe, Tegucigalpa, Honduras; ^6^Department of Surgery, Minnesota Oncology, Minneapolis, Minnesota, USA

## Abstract

**Purpose:** Previous studies have demonstrated that many healthcare workers in low- and middle-income countries (LMICs) lack the appropriate training and knowledge to recognize and diagnose breast cancer at an early stage. As a result, women in LMICs are frequently diagnosed with late-stage breast cancer (Stage III/IV) with a poor prognosis.

**Materials and Methods:** We hosted a 1-day breast cancer educational conference directed towards healthcare workers in Honduras. We conducted pre- and postcourse (1–2 months later) assessments that evaluated knowledge of screening, diagnosis, and treatment of breast cancer. Breast cancer specialists at the University of Minnesota and Honduras developed a 12-question assessment tool in Spanish.

**Results:** A total of 157 people attended the course, and 86 completed the precourse knowledge assessment. The overall percentage of correct responses was 70% in the precourse assessment. Postcourse knowledge assessments were completed by 94 participants. The overall percentage of correct responses was 80% in the postcourse assessment and was significantly higher than precourse assessment scores (*p* < 0.0001). For the individual domains of screening, diagnosis, and treatment, the postcourse knowledge assessment scores were significantly improved as compared with the precourse scores (*p* < 0.0001).

**Conclusion:** In this study, we found that a 1-day, in-person breast cancer educational course directed towards healthcare workers in Honduras resulted in improved breast cancer knowledge assessment scores. Future research and implementation strategies will include training healthcare workers throughout Honduras and determining the impact of these educational interventions on the late-stage presentation of breast cancer.

## 1. Introduction

Breast cancer is the most common cancer globally and is the leading cause of cancer deaths among women worldwide [1]. Breast cancer deaths disproportionately affect women living in low- and middle-income countries (LMICs), where the majority of deaths occur in women younger than 70 years old [2]. In most LMICs, breast cancer is diagnosed at a late stage (Stage III/IV), when treatment is generally less effective, more expensive, and more disabling [3]. Late-stage diagnosis markedly reduces survival, and treatment is both difficult and resource-intensive.

Substantial delays of months or more than a year from the onset of initial breast symptoms to the initiation of breast cancer treatment contribute to late-stage diagnosis and elevated mortality rates in LMICs. The greatest delays to breast cancer diagnosis in LMICs are not attributable to patients delaying care but rather occur after the first medical consultation has taken place [4]. Even when a patient seeks care soon after the onset of symptoms, this does not always translate to early diagnosis and treatment. If the first or subsequent providers do not have the appropriate training or knowledge to recognize early breast cancer and do not know where or how to refer for necessary diagnostic intervention(s), diagnostic delay will result. Previous studies have demonstrated that many healthcare workers in LMICs lack such skills [5]. Effective implementation of early cancer diagnosis programs should include community-based education, with cancer awareness and training, and subsequent deployment of healthcare workers. Successful planning and implementation of such programs, along with robust monitoring and evaluation, are necessary to effectively downstage breast cancer and ultimately improve mortality rates in LMICs.

Honduras is an LMIC with a population of approximately 10 million, and the International Agency for Research on Cancer recently reported that breast cancer is the most commonly diagnosed cancer for Honduran women [6]. Half of the population of Honduras lives in rural and remote areas, with most of these individuals living in poverty [7]. In one study, approximately 60% of rural breast cancer patients in Honduras were diagnosed with an advanced-stage cancer (Stages IIb–IV) [8]. We hosted a 1-day breast cancer educational conference directed towards healthcare workers in Honduras. We conducted pre- and postcourse assessments that evaluated knowledge of screening, diagnosis, and treatment of breast cancer. Our objective was to determine if an educational intervention could improve breast cancer knowledge among healthcare workers in Honduras.

## 2. Methods

**Figure 1:**
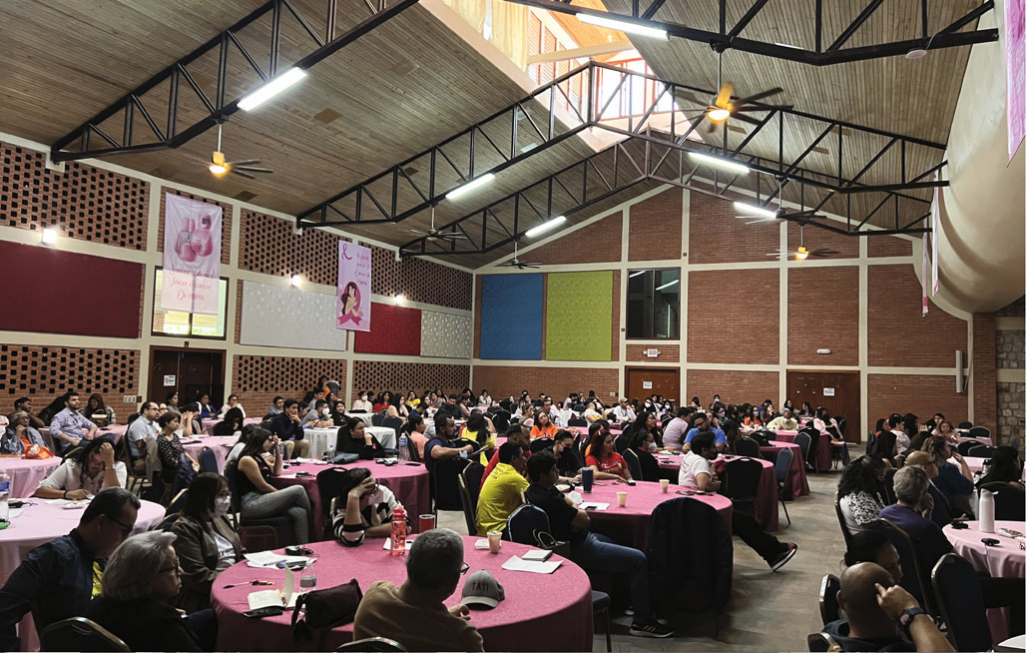
One-day, in-person educational conference in Honduras.

Breast cancer specialists from the University of Minnesota and Honduras organized a 1-day, in-person breast cancer educational course in Honduras in March 2023 (Figure 1). The conference organizers from the University of Minnesota and One World Surgery Honduras sent emails and letters to primary healthcare workers throughout Honduras. We targeted primary care physicians and nurses who see women in Honduras with breast cancer. Most participants were invited through nonprofit organizations/foundations including One World Surgery Honduras, Centro de Cancer Emma Romero de Callejas, Liga Contra El Cancer Honduras, and Sociedad Hondureña de Oncologia. The trainers included radiologists, pathologists, surgeons, and medical oncologists from Honduras and the University of Minnesota. Our primary course objectives were to train healthcare workers to recognize the signs and symptoms of breast cancer and to emphasize the importance of prompt diagnosis and treatment. The format included lectures, hands-on demonstrations, and tumor boards. Topics included breast cancer screening, diagnosis, and treatment. Lectures that were presented in English were translated into Spanish in real time.

This study was approved by the University of Minnesota Institutional Review Board. Informed consent for the study was obtained for all research participants. The course participants were informed that their involvement in the study was voluntary and that they could continue with the course without participation in the research study. We created a 12-question assessment tool in Spanish that evaluated knowledge of breast cancer screening, diagnosis, and treatment. The assessment tool was developed by breast cancer specialists at the University of Minnesota and Honduras and utilized multiple choice and true/false questions. The questions were piloted for content and clarity and tested by 20 people from the United States and Honduras who were not study participants.  Table 1 lists examples of questions in each domain, and the Appendix contains the entire assessment tool. The average time to complete the knowledge assessment was 8–10 min. The precourse knowledge assessment was administered online and onsite just prior to the start of the course. All the precourse questionnaires were administered in person at the course. Most were submitted electronically, while a few were submitted in paper form because of limited internet access by some participants. We found no differences between the paper and electronic baseline scores. One month after the course, we contacted the participants to complete the postcourse knowledge assessment online. Nonresponders were contacted 2 weeks later and then weekly for a total of 4 weeks. All postcourse knowledge assessments were completed 1–2 months after the course and were submitted online. Pre- and postcourse scores for the entire assessment tool and for each domain of screening, diagnosis, and treatment were compared by linear regression. We also collected demographic data of the course participants.

## 3. Results

A total of 157 people attended the course, and 86 completed the precourse knowledge assessment. The median age of the participants was 30 years; 49% had previously undertaken some breast cancer educational activity; 54% worked primarily in a hospital setting; and 40% were physicians (Table 2). The overall percentage of correct responses was 70% in the precourse assessment (Table 3). Employment status (clinic vs. hospital), age, and prior educational activities were not significantly associated with initial baseline knowledge assessment scores (coefficients *β* were 3.84 [*p* = 0.87], 9.65 [*p* = 0.86], and 4.52 [*p* = 0.72], respectively).

Specific questions that had less than 70% correct responses were as follows:

• Frequency of breast screening
• Management of breast erythema
• Estrogen receptor testing
• Chemotherapy for breast cancer
• Prognosis of breast cancer

Postcourse knowledge assessments were completed by 94 participants. The overall percentage of correct responses was 80% in the postcourse assessment and was significantly higher than precourse assessment scores (Table 3). For the individual domains of screening, diagnosis, and treatment, the postcourse knowledge assessment scores were significantly improved as compared with the precourse scores. Age and prior educational activities were not significantly associated with knowledge assessment postcourse scores (coefficients *β* were 0.59 [*p* = 0.22] and 0.66 [*p* = 0.15]); however, employment status (clinic) was associated with increased score *β* 1.35 (*p* = 0.03). Specific questions that persistently had less than 70% correct responses in the postcourse assessment were as follows:

• Frequency of breast screening
• Management of breast erythema
• Estrogen receptor testing

## 4. Discussion

In this study, we found that a 1-day, in-person breast cancer educational course directed towards healthcare workers in Honduras resulted in improved breast cancer knowledge assessment scores 1–2 months later. We found significant improvement in overall scores as well as in screening, diagnosis, and treatment domains. In the postcourse assessment, we found continued knowledge deficits in the frequency of breast screening, management of breast erythema, and estrogen receptor testing.

A substantial proportion of women with breast cancer in LMICs are diagnosed at a late stage (American Joint Committee on Cancer (AJCC) Stage III or IV), ranging from 30% to 50% in Latin America to 75% in sub-Saharan Africa [9, 10]. Patients with breast cancer in LMICs often experience significant delays to diagnosis and treatment even after they present to the healthcare system. The Breast Health Global Initiative recommends that the diagnostic interval that includes clinical evaluation, imaging, and biopsy be completed within 60 days [11]. Survival outcomes are worse if this interval is longer than 3 months [12]. Yet, few LMICs achieve a diagnostic interval within 60 days [13].

Early detection requires that trained healthcare workers recognize the signs and symptoms of breast cancer and understand the impact of delays. Previous studies have demonstrated that many healthcare workers in LMICs lack such skills. In a study from Cameron, Nguefack et al. reported that less than half of the healthcare professionals had good knowledge of breast cancer [14]. In Botswana, many healthcare workers at local clinics did not recognize cancer symptoms and misdiagnosed women as having other common health problems such as sexually transmitted diseases or tuberculosis [5]. In a study of healthcare workers from Nigeria, only 49% of physicians believed that breast cancer could not be cured with herbal or alternative therapies, and 42% thought that breast cancer could completely disappear with prayer [15]. There have been no previous publications evaluating breast cancer knowledge or skills of healthcare workers in Honduras. One of the objectives of this study was to determine the baseline knowledge level of healthcare workers in Honduras. At the precourse knowledge assessment, 70% of the responses were correct. Perhaps these individuals who decided to attend a breast cancer conference already had an interest and baseline level of knowledge of breast cancer and are not representative of healthcare workers in Honduras.

Implementation of programs that focus on training healthcare workers may decrease rates of late-stage breast cancer. Sarawak General Hospital in Malaysia instituted an educational program that included training 400 healthcare workers in early diagnosis techniques including clinical breast examination [16]. Four years after implementation, the proportion of Stage III/IV breast cancer was reduced from 60% to 35%. These results were accomplished without screening mammography. In two Mexican states, Keating et al. reported that competency-based breast cancer education improved the knowledge of community healthcare workers about breast cancer risk factors, signs and symptoms, screening, and treatment [17]. Similarly, Rehman et al. reported that an educational intervention improved health professionals' knowledge about breast cancer risk, presentation, and screening in Pakistan [18]. Pace et al. conducted a breast cancer training program for community healthcare workers in Rwanda that focused on recognizing signs and symptoms of breast cancer, clinical breast evaluation, and navigation; the educational intervention resulted in a higher proportion of early-stage breast cancer [19]. A Cochrane analysis of four randomized clinical trials (two in India, one in Rwanda [cited above], and one in the Philippines) evaluated the effectiveness of training healthcare workers in clinical breast examination in LMICs; the authors concluded that training healthcare workers in clinical breast examination may provide some benefit to early detection [20].

We acknowledge several limitations to this study. The postcourse assessment was completed between 1 and 2 months after the course; it is not clear if breast cancer knowledge will be retained for a longer duration. The follow-up knowledge assessment was completed outside the course venue, so participants could have used the internet or other sources to improve their test scores (“open book” examination). Additionally, the healthcare workers who participated in this course may not be representative of all healthcare workers in Honduras. Finally, improved breast cancer knowledge may not translate into earlier diagnosis and treatment. Despite these limitations, this study demonstrates that in at least one LMIC, breast cancer knowledge among healthcare workers can be improved with a 1-day educational course.

In summary, we found that a 1-day educational course improved breast cancer knowledge among healthcare workers in Honduras. To our knowledge, this is the first report of an intervention that improved breast cancer knowledge in Central America. We think that the differences in knowledge scores are clinically meaningful, but the actual proof will be if such educational activities result in meaningful decreases in advanced-stage breast cancer. The persistence of knowledge deficits on the postcourse assessment may highlight the need for multiple educational interventions over time. Future research and implementation strategies will include training healthcare workers throughout Honduras and determining the impact of these educational interventions on the late-stage presentation of breast cancer.

## Figures and Tables

**Table 1:** Examples of knowledge-assessment questions for the three domains.

**Screening**
For an average-risk woman, how often should breast screening be performed?
**Diagnosis**
Which of the following can be a sign or symptom of breast cancer?
**Treatment**
Chemotherapy is recommended for all women with breast cancer.

**Table 2:** Characteristics of healthcare workers.

Healthcare degree	
Physicians	31%
Nurses	19%
Pharmacy, others	44%
Did not answer	6%
Gender	
Female	71%
Male	29%
Type of employment	
Clinic-based	35%
Hospital-based	42%
Others	23%
Age	
< 18 years	0%
19–30 years	50%
31–40 years	42%
41–50 years	3%
51–60 years	3%
> 60 years	1%
Prior breast cancer education	
Yes	49%
No	51%
Years of practicing since training	
0–3 years	67%
4–10 years	27%
11–20 years	3%
> 20 years	3%
Number of women seen/month with breast complaint	
0–10	82%
11–20	15%
> 20	3%
Area of practice	
General medicine	36%
Surgery	2%
Oncology	5%
Gynecology	2%
Others	54%

**Table 3:** Proportion of correct responses to 12-question breast cancer knowledge assessment.

	**Precourse**	**Postcourse**	**p** ** value**
Overall	70%	80%	0.0001
Screening	67%	76%	0.0001
Diagnosis	82%	87%	0.0001
Treatment	62%	79%	0.0001

## Data Availability

The data that support the findings of this study are available on request from the corresponding author. The data are not publicly available due to privacy or ethical restrictions.

## Appendix A: Conferencia Cancer De Mama

March 11, 2023

Knowledge assessment (% correct precourse/% correct postcourse)

1. The risk of developing breast cancer increases with (73%/89%):
  a. Increasing woman's age Y/N/I don't know
  b. Having a sister with breast cancer Y/N/I don't know
  c. Any alcohol use Y/N/I don't know
  d. Having large breasts Y/N/I don't know
  e. Breast feeding Y/N/I don't know
  f. Sedentary lifestyle Y/N/I don't know
  g. Having first child before age 30 years Y/N/I don't know
  h. Obesity Y/N/I don't know
2. Which of the following statements regarding breast cancer screening is true (79%74%)?
  a. Mammograms involve harmful radiation exposure.
  b. Clinical breast examination is an effective means of breast cancer screening.
  c. Breast cancer screening should start for women at the age of 20 years.
  d. Breast cancer screening is not necessary for women who do not have a family history of breast cancer.
3. The incidence of breast cancer in low- and middle-income countries is increasing (97%/99%). T or F.
4. For an average-risk woman, how often should breast screening be performed (17%/41%)?
  a. Once a month
  b. Twice a year
  c. Every other year
  d. Every 5 years
5. A 55-year-old woman noticed a nonpainful right breast lump 2 months ago. She states that the lump has not grown. On physical examination, she has a 3 cm mobile mass in her right breast. What is the most appropriate next step (81%/84%)?
  a. Surgical excision of the lump
  b. Mastectomy
  c. Return to clinic in 6 weeks for repeat examination
  d. Breast ultrasound
  e. Chest radiograph
6. A 32-year-old woman noticed redness in her left breast for 4 weeks. She has not noticed any fever. She is presently 6 months postpartum and has been breastfeeding. Two weeks ago, she was given oral antibiotics, but the redness has persisted. On physical examination, she has a 4 cm area of erythematous skin with edema. What is the most appropriate next step (62%/69%)?
  a. Change antibiotics and re-evaluate in 2 weeks
  b. Perform an ultrasound
  c. Surgically excise the erythematous skin
  d. Start topical steroids
7. Which of the following can be a sign or symptom of breast cancer (90%/99%)?
  a. Lump in the axilla Y/N/don't know
  b. Breast redness Y/N/don't know
  c. Sagging breasts Y/N/don't know
  d. Breast pain Y/N/don't know
  e. Loss of menstruation Y/N/don't know
  f. Puckering of the nipple Y/N/don't know
  g. Bloody nipple discharge Y/N/don't know
  h. Bilateral hand pain Y/N/don't know
  i. Change in breast size Y/N/don't know
8. When detected early, breast cancer is curable (95%/100%). T or F.
9. Mastectomy is recommended for all women with breast cancer (71%/84%). T or F.
10. A 62-year-old woman is diagnosed with a 3 cm, estrogen receptor–negative breast cancer. Which of the following would not be a recommended treatment (45%/62%)?
  a. Tamoxifen
  b. Mastectomy
  c. Chemotherapy
  d. Lymph node removal
11. Chemotherapy is recommended for all women with breast cancer (62%/74%). T or F.
12. The survival of women with breast cancer is worse when (choose Y/N/do not know) (63%/91%):
  a. The cancer has spread to the lymph nodes
  b. The cancer is larger
  c. The cancer is in the right breast (compared to the left)
  d. The diagnosis is delayed by more than 2 months from the time of first provider consultation
  e. The cancer was diagnosed by needle biopsy (compared to open biopsy)
